# A Cerebral Hemorrhage Revealing a Cerebral Venous Thrombosis

**DOI:** 10.7759/cureus.103136

**Published:** 2026-02-06

**Authors:** Hicham Hammadi, Ilias Hjaoua, Ouadie Elmenaoui, Houmed Houssein Houmed, Mourad Ababou, Noureddine Kartite, Anas Elbouti, Abdelhafid Houba, Bakkali Hicham, Nawfal Doghmi

**Affiliations:** 1 Anesthesiology and Reanimation, Military Hospital Mohammed V, Rabat, MAR; 2 Radiology, Military Hospital Mohammed V, Rabat, MAR

**Keywords:** anticoagulation, cerebral, hemorrhage, thrombosis, women

## Abstract

Cerebral venous thrombosis (CVT) is a rare neurovascular disorder that primarily affects young women, largely due to hormonal risk factors such as oral contraceptive use and pregnancy. We report the case of a 31-year-old woman in whom CVT presented with an intracerebral hemorrhage. Therapeutic anticoagulation with low-molecular-weight heparin (LMWH) was initiated without complications, resulting in a favorable clinical outcome. This report highlights the importance of maintaining a high index of diagnostic suspicion for CVT in young patients with risk factors and reinforces the counterintuitive but guideline-supported principle of anticoagulation even in the presence of intracerebral hemorrhage. The diagnosis was confirmed by contrast-enhanced CT. This report adds novelty by demonstrating successful management in this context and provides a concise educational example for clinicians.

## Introduction

Cerebral venous thrombosis (CVT) is defined as thrombosis of the dural venous sinuses and/or cerebral veins [1]; it has an estimated incidence of approximately 12 cases per million inhabitants per year, with higher rates reported in low- and middle-income countries [2,3]. In young women, major risk factors include oral contraceptive use and pregnancy or the puerperium [4,5]. CVT can occasionally present with an intracerebral hemorrhage [1], a presentation that is clinically challenging and often misdiagnosed, highlighting the need for a high index of diagnostic suspicion in patients with risk factors. Management of CVT relies on therapeutic anticoagulation, even in the presence of hemorrhage, in accordance with current guidelines. Clinically, CVT typically presents with intracranial hypertension, focal neurological deficits, and seizures [1]. We report a case of CVT presenting with an intracerebral hemorrhage.

## Case presentation

**Figure 1:**
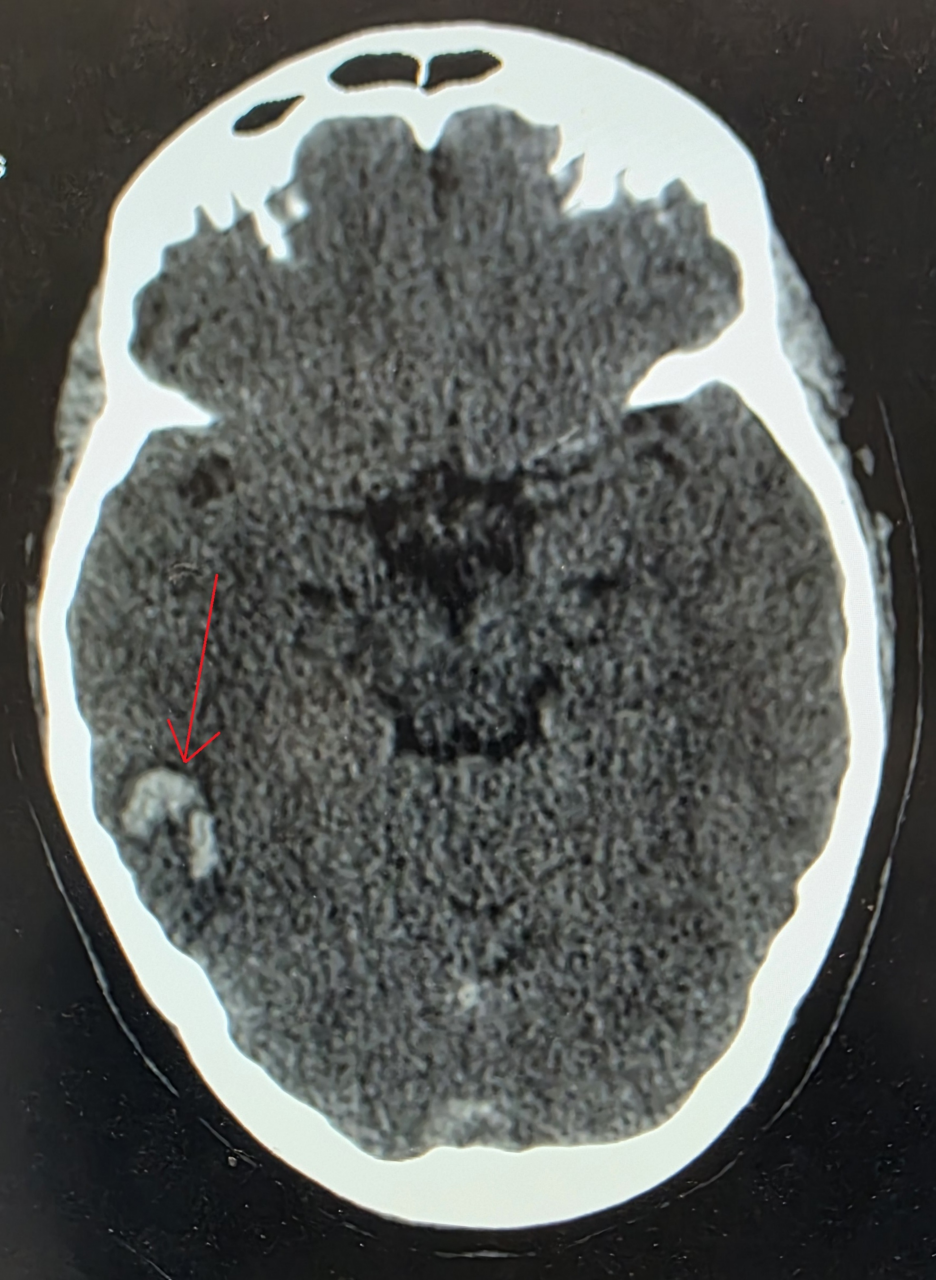
Non-contrast axial brain CT showing a right temporal hemorrhagic infarction (arrow) CT: computed tomography

A 31-year-old female was admitted with impaired consciousness. She had no prior medical or surgical history. She was gravida 2, para 2, with two living children aged eight and six years. The patient had been using oral contraceptives for six years, specifically combined estrogen-progestin pills containing ethinylestradiol 30 µg and drospirenone 3 mg. The history of the present illness dated back to 24 hours before admission, beginning with a progressively worsening headache. The symptoms intensified and were not associated with nausea or vomiting. On admission, clinical examination revealed a confused patient with a Glasgow Coma Scale score of 13, due to an altered verbal response. Pupils were equal and reactive, and no sensory or motor deficits were noted. The patient subsequently experienced a generalized tonic-clonic seizure lasting less than five minutes. She was hemodynamically and respiratorily stable, afebrile, and had no signs of meningeal irritation. Laboratory investigations (Table 1) showed no abnormalities. Blood glucose was 1.34 g/L, inflammatory markers were negative, and metabolic, electrolyte, and phosphocalcium panels were all within normal ranges. A non-contrast brain CT scan revealed a right temporal intracerebral hemorrhage (Figure 1).

**Table 1:** Laboratory findings at admission

Parameter	Value	Reference range
Hemoglobin	12 g/dL	12 - 16 g/dL
White blood cell count	8,500/mm³	4,000 - 10,000/mm³
Platelet count	325,000/mm³	150,000 - 450,000/mm³
Prothrombin time	80%	70% - 100%
C-reactive protein	4 mg/L	<5 mg/L
Glucose	1.34 g/L	0.7 - 1.1 g/L
Sodium	141 mmol/L	135 - 145 mmol/L
Potassium	3.8 mmol/L	3.7 - 5.3 mmol/L
Magnesium	1.84 mg/dL	1.6 - 2.2 mg/dL
Phosphate	35 mg/L	25 - 50 mg/L
Calcium	90 mg/L	80 - 105 mg/L
Urea	0.2 g/L	0.15 - 0.5 g/L
Creatinine	8 mg/L	7-12 mg/L

**Figure 2:**
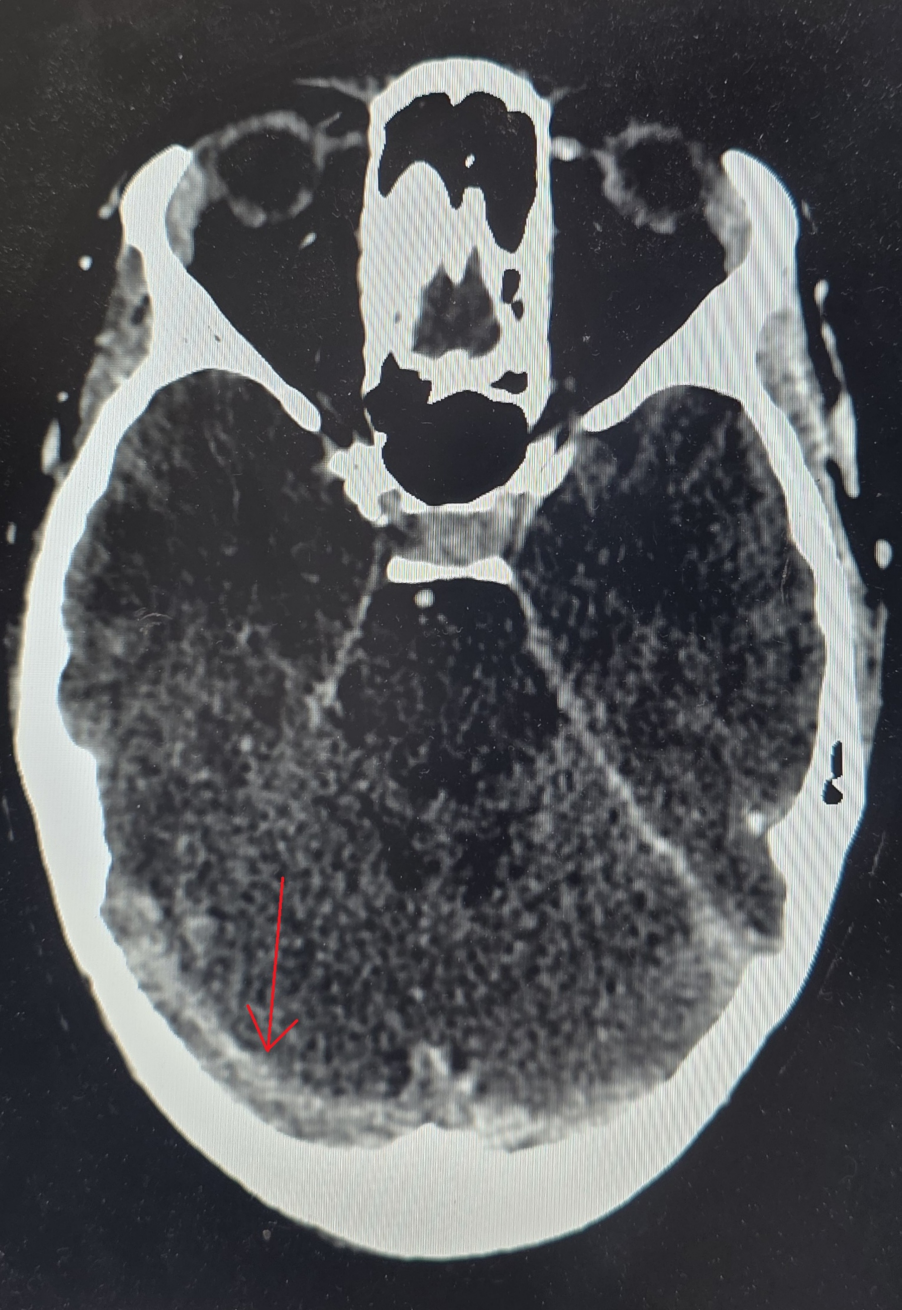
Cerebral CT angiography showing cerebral venous thrombosis of the right transverse sinus (arrow) CT: computed tomography

**Figure 3:**
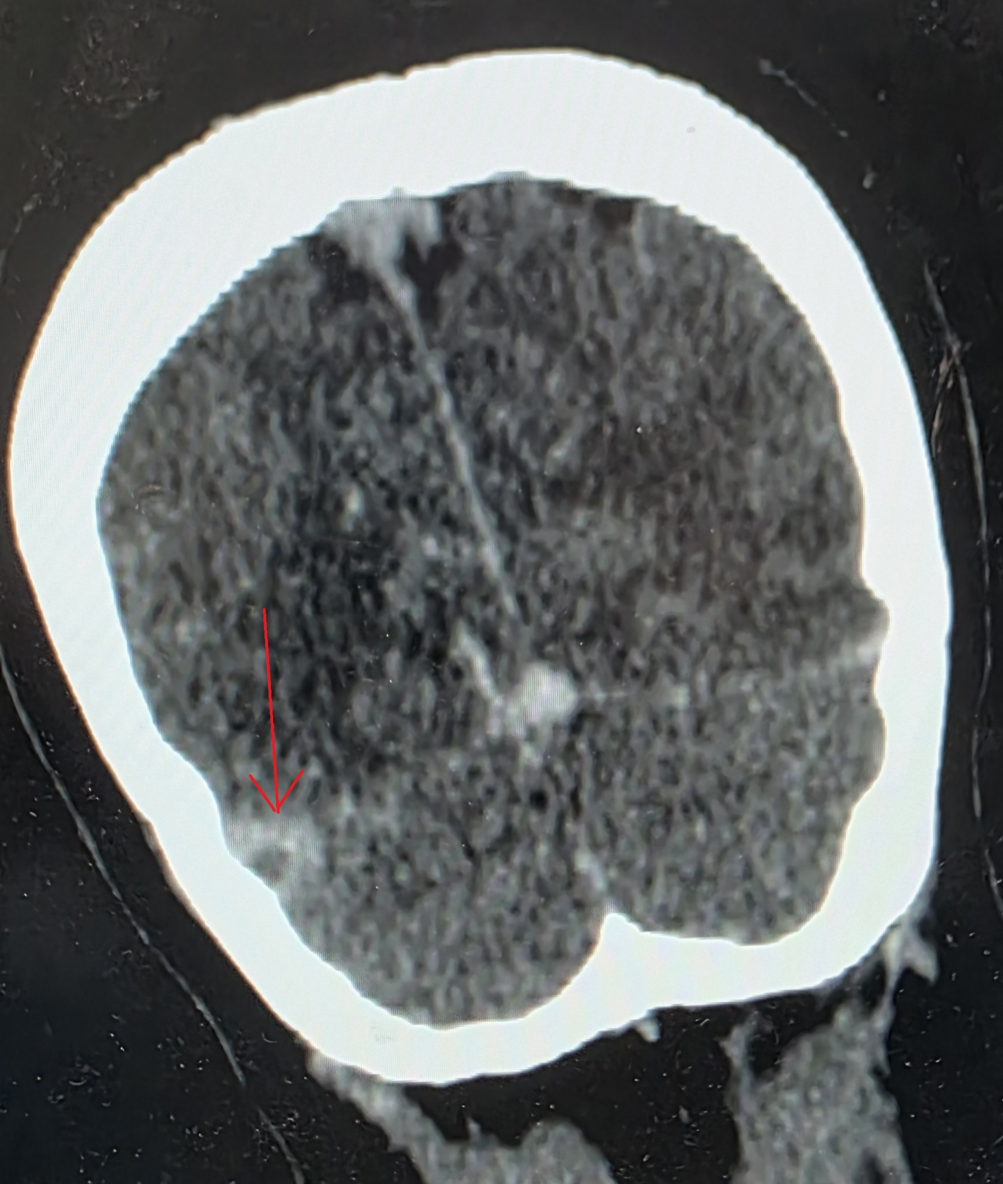
Contrast-enhanced axial brain CT showing the empty delta sign (arrow) CT: computed tomography

Contrast-enhanced CT (Figures 2, 3) demonstrated thrombosis of the right transverse sinus. The patient was admitted to the ICU for monitoring and management. Therapeutic anticoagulation with enoxaparin 80 mg every 12 hours was initiated, along with antiepileptic treatment consisting of levetiracetam 500 mg every 12 hours and clobazam 5 mg every 12 hours. Gastric protection with omeprazole was also provided. The clinical course was favorable, marked by progressive neurological improvement leading to complete recovery. Three days after admission, the patient was transferred to the neurology department for ongoing management. Thrombophilia testing is planned after the resolution of the acute phase and temporary cessation of anticoagulation to ensure accurate and reliable results. The patient’s functional outcome was excellent, with full restoration of her baseline neurological status.

## Discussion

CVT is defined as thrombosis of the dural venous sinuses and/or cerebral veins [1]. Its average incidence is estimated at approximately 12 cases per million inhabitants per year, with higher rates reported in low- and middle-income countries [2,3]. CVT affects women approximately three times more often than men, particularly during the reproductive years, with a median age of onset in the fourth decade of life [6]. However, recent studies from the United States and India suggest a rising incidence among older patients and men [2,7].

Most patients with CVT present with one or more risk factors, whether transient or chronic. In young women, oral contraception and pregnancy or puerperium are the main contributors [4,5]. Other well-recognized factors include acquired thrombophilias (antiphospholipid syndrome, JAK2 mutations, malignancies, and autoimmune diseases such as Behçet’s disease and inflammatory bowel disease) and inherited thrombophilias (protein C or S deficiency, factor V Leiden, and the prothrombin G20210A mutation) [8]. Transient risk factors include infections (ENT infections and COVID-19), dehydration, certain medications (corticosteroids and L-asparaginase), and vaccine-induced thrombotic thrombocytopenia [8]. Mechanical factors, such as head trauma, neurosurgery, or compressive lesions, may also contribute [8].

The etiopathogenesis of CVT is based on the interaction of the three components of Virchow’s triad: blood hypercoagulability, endothelial injury, and abnormalities in blood flow [9]. Depending on the patient’s individual risk factors, one mechanism may predominate [10]. The most common signs include headache (observed in more than 80% of cases), focal deficits, generalized or partial seizures, papilledema, and altered consciousness ranging from confusion to deep coma [11]. Clinical presentation can be acute (less than 48 hours), subacute (up to 30 days), or chronic (more than 30 days). Gradual development of intracranial hypertension may occur in isolation and result from increased intracranial vascular volume due to impaired venous outflow or from impaired cerebrospinal fluid absorption when the Pacchioni granulations are involved [12]. Epileptic manifestations occur in approximately 40% of cases during the initial phase and in about 7% of cases within two weeks of diagnosis [11].

In suspected CVT, brain MRI is the gold standard for diagnosis, while CT remains widely used due to its accessibility. Contrast-enhanced CT is necessary to confirm or exclude CVT [13], though it may appear normal in 4 to 25% of patients, particularly in cases of isolated intracranial hypertension [14]. Direct CT signs include thrombus hyperdensity (the “cord sign” or “dense triangle”), with the delta sign on contrast-enhanced scans seen in 30 to 46% of cases [13]. Indirect findings reflect the effects of venous occlusion, such as cerebral edema (approximately 50%), hemorrhagic venous infarctions, and abnormal cortical, falx, or tentorial enhancement indicating collateral circulation [15]. Standard MRI sequences include T1, T2, and FLAIR imaging, with MR venography recommended when diagnostic uncertainty persists [15]. Cerebral angiography is now reserved for select cases due to its invasive nature and the high diagnostic performance of MRI and CT [13].

Management of CVT relies on anticoagulation, even in the presence of intracerebral hemorrhage, to limit thrombus propagation, promote recanalization, and prevent recurrent venous thromboembolism. Guidelines from the American Heart Association/American Stroke Association and European societies recommend low-molecular-weight heparin (LMWH) rather than unfractionated heparin during the acute phase, followed by a transition to vitamin K antagonists (VKAs) for 3-12 months in cases of transient risk factors, or long-term in the presence of persistent major risk factors [1,16,17]. LMWH is preferred due to its ease of administration, predictable anticoagulant effect, lower risk of thrombocytopenia, and association with better clinical outcomes [18,19]. The occurrence of venous hemorrhage is not considered a contraindication to anticoagulation [1,16,20]. Finally, direct oral anticoagulants (DOACs), whose efficacy and safety have been established in deep vein thrombosis and pulmonary embolism, also appear to be a viable therapeutic alternative for CVT [8].

DOACs are contraindicated in pregnant women (both DOACs and VKAs are contraindicated; only LMWH is recommended) and in breastfeeding women. DOACs are also associated with a higher risk of thromboembolic recurrence compared with warfarin in patients with antiphospholipid syndrome [21,22]. Based on currently available data, it is reasonable to transition to DOACs or VKAs after an initial period of parenteral anticoagulation. However, it is not yet established whether a five- to 15-day course is safer or more effective than a shorter period [23]. Currently, endovascular treatment (EVT) is primarily reserved as a rescue option for patients with clinical deterioration, treatment failure, or contraindications to standard therapy [8]. Decompressive craniotomy should be considered for patients with severe acute CVT associated with parenchymal lesions and an imminent risk of brain herniation, as a life-saving therapeutic approach [8].

This report highlights the link between oral contraceptive use as a predisposing factor, CVT, and secondary intracerebral hemorrhage, while demonstrating a favorable response to anticoagulation. A thrombophilia workup will be conducted after the acute phase to rule out any underlying disorders that could affect the duration of treatment, further reinforcing the application of evidence-based principles to individualized patient care.

## Conclusions

CVT remains a rare condition with a highly variable clinical presentation, which can occasionally manifest as an intracerebral hemorrhage, as illustrated in our case. This atypical presentation underscores the importance of maintaining a high index of diagnostic suspicion for CVT in any unexplained cerebral hematoma, particularly in young patients with risk factors. Therapeutic anticoagulation, even in the presence of hemorrhage, remains the cornerstone of treatment and a key determinant of prognosis. In our patient, early recognition and timely anticoagulation resulted in a favorable outcome, highlighting the practical benefit of prompt diagnosis and appropriate management.
